# Supplementary material for: High caseload of Scabies amongst Rohingya refugees in Cox’s Bazar, Bangladesh: A retrospective analysis of the epidemiological and clinical characteristics of cases, July 2022 to November 2023
Source: PLOS Glob Public Health. 2025 Apr 9;5(4):e0003981. doi: 10.1371/journal.pgph.0003981 (PMC11981167; doi:10.1371/journal.pgph.0003981)
Supplement: Attachment — Submitted filename: Response to Reviewers_PGPH-D-24-01980R1.docx [file pgph.0003981.s002.docx]

**Response to Reviewers -** PGPH-D-24-01980R1

Dear Editor and reviewers,

Thank you very much for providing your invaluable review to our article entitled " **High caseload of Scabies amongst Rohingya refugees in Cox’s Bazar, Bangladesh: A retrospective analysis of the epidemiological and clinical characteristics of cases, July 2022 to November 2023**". We carefully read the comments and strive to address them appropriately.

We are looking forward receiving your final decision.

With kindest regards.

**Temmy Sunyoto (corresponding author)**

**Comments and Answers**

| **Reviewer 1 comments** | **Answer** |
| --- | --- |
| Overall, the manuscript is well-written and organized, but there are a few areas where clarification and additional discussion could strengthen the paper.  The introduction provides and adequate background but please provide more in-depth context about the importance of addressing scabies in refugee settings, emphasizing the public health and human rights aspects of this issue. | We thank the reviewer for the overall assessment of our draft.  For introduction section, we have now added these in the Introduction Line 61-65 (with references) to be able to your excellent point:  *“In these vulnerable populations, untreated scabies leads to severe complications, increasing morbidity and burdening already limited healthcare. The intense itching and skin damage caused by scabies significantly reduce quality of life, adding to the daily suffering of affected individuals. Addressing* scabies *is thus a critical public health need and a human rights imperative, as these populations deserve basic healthcare and protection from preventable disease”* |
| The manuscript provides detailed information about the treatment regimens used (i.e., ivermectin and permethrin). However, more discussion about the limitations of these treatments, particularly in the context of limited water access for permethrin application, would be valuable. | We noted this comment and we have added more information about the limitations of the treatment has been included in the discussion **Line 232-236**  *Although its application does not strictly require bathing beforehand, the recommendation to apply permethrin on all skin surfaces and remove it after 8–14 hours through washing may pose significant difficulties without adequate water resources (32). In water-scarce settings, thorough cleansing post-treatment might not be feasible, which could reduce treatment efficacy, increase the risk of residual irritation, or complicate hygiene practices.*  And also for line **255-261** (in MDA section) we have added the logistic and cost constrain for using permethrin, and also the challenge in adhering to application guidelines which requires water/soap and change of clothes. |
| The paper also briefly mentions antibiotic use for complicated cases, but further detail on the types of bacterial infections treated and the outcomes for those patients would enrich the findings. | More information about the use of antibiotic in complicated cases has been included in the discussion (**Line 244-248**)  *“In cases of scabies complicated by bacterial infections, standard treatment for impetigo*  *includes topical application of mupirocin for localized infections and oral antibiotics, such as cefalexin or cloxacillin, for more extensive or complicated cases. In neonates with lesions around the umbilicus, intravenous cloxacillin is administered to mitigate the risk of systemic infection (24). Although these regimens aim to effectively manage scabies-related bacterial complications, we unfortunately lack follow-up data to confirm treatment outcomes in this cohort”.* |
| Expand the discussion on the challenges and future directions for interventions in similar refugee settings. Specifically, consider elaborating more on the WASH interventions and any gaps in community health education that might need to be addressed. | The discussion around the key challenges and limitations has been expanded – see Line **290-292**  *“Effective MDA requires strong community engagement; in Cox’s Bazar, health promotion efforts and risk communication were tailored to the specific context, with the involvement of community health workers and camp leaders proving indispensable.”*  *Also line* ***300-305*** *: This study underscores the challenges of managing scabies outbreaks in camps where inadequate WASH infrastructure, limited access to clean water, and poor sanitation persist. Strengthening WASH interventions, including the provision of functional washing facilities, soap, and well-maintained latrines, is crucial. Coupled with appropriate community health education, these improvements could significantly enhance scabies control efforts in these vulnerable settings. Prior endemicity of scabies in the host communities need to be considered too.* |
| Address the limitations in a more structured way, including any biases or confounding factors that may have impacted the study results. | The limitation has been updated as per suggestion and we hope now reads better  See Line 306-319. |
| **Reviewers 2 comments** | **Answers** |
| Cox Bazar vs Cox's Bazar – please review for consistency throughout | Thanks a lot for noticing this, I made sure to fix this throughout the manuscript. |
| Discordance of stated population numbers – three different numbers are mentioned in relation to the population (as well as only two camps vs more than two camps). This does get confusing at times, please clarify. Specifically, the abstract, the methods, and the results differ in number. | We have now carefully review where the population numbers appear in our manuscript and ensure their consistency. All previous information has been revised and unified to avoid any confusion. |

**Other edits to not forget about**

Journal Requirements:

1. Some material included in your submission may be copyrighted. According to PLOS’s copyright policy, authors who use figures or other material (e.g., graphics, clipart, maps) from another author or copyright holder must demonstrate or obtain permission to publish this material under the Creative Commons Attribution 4.0 International (CC BY 4.0) License used by PLOS journals. Please closely review the details of PLOS’s copyright requirements here: PLOS Licenses and Copyright. If you need to request permissions from a copyright holder, you may use PLOS's Copyright Content Permission form.

Please respond directly to this email or email the journal office and provide any known details concerning your material's license terms and permissions required for reuse, even if you have not yet obtained copyright permissions or are unsure of your material's copyright compatibility.

**Potential Copyright Issues:**

**Figure 1:** please (a) provide a direct link to the base layer of the map (i.e., the country or region border shape) and ensure this is also included in the figure legend; and (b) provide a link to the terms of use / license information for the base layer image or shapefile. We cannot publish proprietary or copyrighted maps (e.g. Google Maps, Mapquest) and the terms of use for your map base layer must be compatible with our CC-BY 4.0 license.

Note: if you created the map in a software program like R or ArcGIS, please locate and indicate the source of the basemap shapefile onto which data has been plotted.

If your map was obtained from a copyrighted source please amend the figure so that the base map used is from an openly available source. Alternatively, please provide explicit written permission from the copyright holder granting you the right to publish the material under our CC-BY 4.0 license.

Please note that the following CC BY licenses are compatible with PLOS license: CC BY 4.0, CC BY 2.0 and CC BY 3.0, meanwhile such licenses as CC BY-ND 3.0 and others are not compatible due to additional restrictions.

If you are unsure whether you can use a map or not, please do reach out and we will be able to help you. The following websites are good examples of where you can source open access or public domain maps: 
* U.S. Geological Survey (USGS) - All maps are in the public domain. ([http://www.usgs.gov](http://www.usgs.gov/)) 
* PlaniGlobe - All maps are published under a Creative Commons license so please cite “PlaniGlobe, [http://www.planiglobe.com](http://www.planiglobe.com/), CC BY 2.0” in the image credit after the caption. (<http://www.planiglobe.com/?lang=enl>) 
* Natural Earth - All maps are public domain. (<http://www.naturalearthdata.com/about/terms-of-use/>)

**Answer to the editor:**

We are currently checking this with the GIS unit that has provided us with the map based on our request. We shall respond to this mail directly to ensure the above is addressed.

Please review your reference list to ensure that it is complete and correct. If you have cited papers that have been retracted, please include the rationale for doing so in the manuscript text, or remove these references and replace them with relevant current references. Any changes to the reference list should be mentioned in the rebuttal letter that accompanies your revised manuscript. If you need to cite a retracted article, indicate the article’s retracted status in the References list and also include a citation and full reference for the retraction notice.

**Answer to editor: References list has been checked**
