# Supplementary material for: High caseload of Scabies amongst Rohingya refugees in Cox’s Bazar, Bangladesh: A retrospective analysis of the epidemiological and clinical characteristics of cases, July 2022 to November 2023
Source: PLOS Glob Public Health. 2025 Apr 9;5(4):e0003981. doi: 10.1371/journal.pgph.0003981 (PMC11981167; doi:10.1371/journal.pgph.0003981)
Supplement: Attachment — Submitted filename: Response to Reviewers_PGPH-D-24-01980R2.docx [file pgph.0003981.s003.docx]

**Response to Reviewers -** PGPH-D-24-01980R2

Dear Editor and reviewers,

Thank you for continuing to provide valuable review to our article *“****High caseload of Scabies amongst Rohingya refugees in Cox’s Bazar, Bangladesh: A retrospective analysis of the epidemiological and clinical characteristics of cases, July 2022 to November 2023***". We carefully read the comments and strive to address them appropriately.

We are looking forward receiving your final decision.

With kindest regards,

On behalf of the team

**Dr Temmy Sunyoto (corresponding author)**

| **Comments** | **Response** |
| --- | --- |
| I think being very clear about what messages you want to get across with this analysis is important. They could be made more strongly. eg. Scabies is a huge problem in displaced persons camps particularly in the young. Cross contamination and lack of co-ordinated response contribute to ongoing high case loads and epidemic levels of infestation. Co-ordinated management of contacts is crucial to gain control. Not managing scabies outbreaks has considerable potential to long term adverse health outcomes and even mortality The local resident population also needs to be considered and need to benefit from services being provided for displaced persons (you mention this but do not emphasise this is an important consideration) You many want to bring out other aspects. | We thank the reviewer for these sharp observations on the key messages that needs to get across from this work.  We agree with these points, and you see we make our last paragraphs in the manuscript to more strongly reflect these very pertinent points. |
| Abstract: Small point but you indicated scabies is classified as a neglected disease. By whom and where is the reference. I think you mean classified by WHO as a neglected tropical disease….. What is “complicated/sur-infected” do you mean super-infected or impetiginized? If so suggest simplifying and just stating this. | Thank you for this comment.  -Yes, the classification of Neglected Tropical Disease (NTD) is done by the WHO- see [here](https://www.who.int/news-room/fact-sheets/detail/scabies), excerpt: “*In 2017, scabies and other ectoparasites were included as NTDs by the WHO, in response to requests from Member States and the recommendations of the WHO Strategic and Technical Advisory Group for NTDs.*  - Correct for the ‘complicated/sur-infected’ – we are now simplifying it to ‘secondary infection’ |
| Introduction: It is not that rare for the downstream complications of scabies such as glomerulonephritis and I think it is the key message for emphasizing the importance of addressing scabies in the risk to long term health sequele. For instance, symptomatic acute glomerulonephritis was reported in 10% of children in a survey in northern Australia, but, in addition, 24% had microscopic haematuria L Streeton, JN Hanna, RD Messer An epidemic of acute post‐streptococcal glomerulonephritis among Aboriginal children J Paediatr Child Health, 31 (2008), pp. 245-248  You introduce the term mass drug administration in your introduction and the indications for when this would be recommended but then express the results that meant you did not actually use it. So suggest moving this aspect/point to the discussion area. It is already there, I am not clear it required in the introduction. | We concur with this comment – hence we explained this detail in the first paragraph, and we added the suggested reference. We have modified the sentence on line 53-54as well: *emphasizing the need for prompt treatment to mitigate long-term health sequelae.*  For your comment on MDA, we would like to explain that initially we did not mention this in the introduction and touched upon it in the discussion. However, as in the background of this research, advocacy for MDA was a key activity and the point of mentioning it in the introduction is twofold: 1) to indicate that at public health level, an MDA is necessary for this magnitude of an outbreak; and 2) not to give surprise that we discussed MDA at a later section of the paper.  But after careful re-reading, we see that the MDA paragraph can indeed be moved to discussion section and no longer in the introduction. |
| Methods What are your locally adapted health messages given by the community health volunteers? This might be useful to share ( even if included as an addendum)  The term “complicated case” just refers to bacterial infection is that correct? It is not quite clear – if this is the case I suggest you just say impetiginized scabies or secondarily infected scabies. You do indicate in the methods that “simple” means “not infected” - once you have clarified this it will need to be consistently expressed in the manuscript. | The health messages related to scabies were developed by the MSF team and printed/used locally, using the local language – they are mostly not digitalized, but we are happy to share if deemed useful at a later stage.  For the term complicated cases, we have made changes throughout to be ‘impetiginized scabies’ or secondary infection. And indeed, you correctly noted that simple scabies means ‘not infected’. We have reviewed the whole manuscript and made it consistent. |
| Management of scabies: you don’t indicate what protocol you used for contacts of the cases is it possible to state and do you have information on the number of contacts treated per case?  In the results section you indicate that 20% of those treated were contacts – this is low – ie suggesting that the majority of contacts were not treated which would probably explain why there was so little impact on the continuing high level of cases….. Also I suspect there is significant population exchange between the various camps which will contribute to the ongoing spread of cases | We referenced MSF clinical guidelines (MSF Clinical guidelines -March 2023. Available from: [Scabies \| MSF Medical Guidelines](https://medicalguidelines.msf.org/en/viewport/CG/english/scabies-16689646.html))   - Treatment for contacts is the same (with ivermectin or permethrin). Unfortunately, we did not collect date of the number of contact treated per cases, though we included one variable on size of household . As mentioned in the method, clinical cases are encouraged to bring members of their household to the clinic and if they are asymptomatic, they are recorded as ‘contact’. - Therefore the 20% proportion of ‘contacts’ among all treated cases here then refers to the asymptomatic household members who came to the clinic and received treatment. However, we agree that it is likely that many contacts were not treated and this contribute to the continuing high cases. - Population exchange between various camps officially is not allowed (restriction of movement between camps imposed to camps habitants) but in reality, for sure it is impossible to ‘control’, hence we add this element into discussion. |
| Discussion 217: you indicate that such high outbreak levels have not been previously reported and yet you reference the Ethiopian outbreak where there were 379,000 confirmed cases of scabies was identified the Amhara Ethiopian outbreak Enbiale W, Ayalew A. Investigation of a Scabies Outbreak in Drought-Affected Areas in Ethiopia. Trop Med Infect Dis. 2018 Oct 29;3(4):114. doi: 10.3390/tropicalmed3040114. PMID: 30380650; PMCID: PMC6306922. Also the authors went onto administrer MDA Enbiale W, Baynie TB, Ayalew A, Gebrehiwot T, Getanew T, Ayal A, Ayalew M, De Vries HJ, Takarinda K, Manzi M, Zachriah R. "Stopping the itch": mass drug administration for scabies outbreak control covered for over nine million people in Ethiopia. J Infect Dev Ctries. 2020 Jun 29;14(6.1):28S-35S. doi: 10.3855/jidc.11701. PMID: 32614793. This would be relevant to reference and discussion in your context. | Thank you for the comment, indeed we included the Ethiopian experience with large scabies outbreak – BUT, clearly a different context than Bangladesh Cox Bazar – in terms of open versus close settings, as the Ethiopian experience targeted nation wide MDA thus treating whole population, while in CXB, the refugee confinement in the camps are clearly separated from the local communities. WE rephrased the sentence in Line 217.  We take this into account and we have refined our discussion to make this distinction clearer – and we agree that the papers you shared are very relevant. |
| 224: what environmental decontamination measures were conducted? What is the evidence of benefit anyway for such an approach? Suggest you include  261 “Scabies outbreaks, although not attributing to mortality cause significant added burdens on 262 populations including physical and mental distress” I disagree, scabies has an associated mortality – important not to underplay in such a setting.  Pyoderma, chronic renal disease leading to life long hypertension and increased heart disease…. See figure in Toward the Global Control of Human Scabies: Introducing the International Alliance for the Control of Scabies \| PLOS Neglected Tropical Diseases (https://journals.plos.org/plosntds/article?id=10.1371/journal.pntd.0002167)  Which illustrates modelled mortality data for sca290: scabies does have a mortality see above….so don’t underplay this.  I hope these comments are useful to enhance the key messages arising from this analysis. | We have now expanded the environmental decontamination: in the madrasah, MSF team supports in washing and treat their beddings and clothing, also spraying the structure with chlorine solution. There is an internal report on this which unfortunately did not demonstrate a lasting impact  Thank you so much for reminding us the important consequence of scabies also in terms of mortality, we have rephrase these lines (and include the reference) to not undermine the mortality that clearly can stem from scabies.  *Scabies outbreaks, far from being benign, are associated with mortality and impose significant health burdens—including severe physical and mental distress, pyoderma, chronic renal disease leading to lifelong hypertension, and an increased risk of heart disease* |
